# PReS-FINAL-2190: Validation of the new histopathological classification of anca glomerulonephritis and its association with renal outcomes in a paediatric population

**DOI:** 10.1186/1546-0096-11-S2-O25

**Published:** 2013-12-05

**Authors:** M Twilt, D Noone, W Hayes, P Thorner, S Benseler, R Parekh, R Laxer, D Hebert

**Affiliations:** 1 Rheumatology, Hospital for Sick Children, Toronto, Canada; 2 Rheumatology, Birmingham children's hospital, Birmingham, UK; 3 Nephrology, the Hospital for Sick Children, Toronto, Canada; 4 Nephrology, the Hospital for Sick Children, Toronto, Canada; 5 Pathology, the Hospital for Sick Children, Toronto, Canada; 6 Reumatology, the Hospital for Sick Children, Toronto, Canada; 7 Rheumatology, the Hospital for Sick Children, Toronto, Canada; 8 Nephrology, the Hospital for Sick Children, Toronto, Canada

## Introduction

Anti-Neutrophil Cytoplasmic Antibody (ANCA) associated glomerulonephritides (ANCA GN) include granulomatosis with polyangiitis (GPA), microscopic polyangiitis (MPA) and Churg-Strauss. A novel histopathologic classification for GPA and MPA has been proposed and validated in adult populations and found to be predictive of long-term renal outcome.

## Objectives

We aimed to validate this classification system in a paediatric population and identify clinical predictors of renal outcome.

## Methods

We performed a retrospective review of all patients with ANCA GN, diagnosed and followed up until transfer to an adult centre at the Hospital for Sick Children between 1987 and 2012. Renal biopsies were reviewed by a pathologist blinded to patient outcome and classified using the new histopathological classification system of focal, crescentic, mixed, sclerotic and globally sclerotic groups. We determined time to CKD by estimated glomerular filtration rate (eGFR) with repeated creatinine measures using the Schwartz equation, or end stage kidney disease as defined as dialysis dependant. Survival and linear regression analyses were conducted.

## Results

The study population consisted of 42 children (69% male) with ANCA GN (21 GPA, 21 MPA) with a median age of 11.96 (+3.52) years and eGFR 36.6 mls/min/1.73 m^2 ^(IQR 15-87.4). Of the 40 patients with a renal biopsy at time of initial diagnosis, 12 (30%) had focal lesions, 20 (50%) crescentic, 3 (7.5%) mixed, 5 (12.5%) sclerotic and no globally sclerotic lesions. 13 (31%) patients required dialysis at baseline. Survival analysis of time to the composite renal endpoint of at least 3 months of eGFR < 60 ml/min/1.73 m^2 ^or ESKD differed among all 3 biopsy groups [p (logrank) = 0.0001; figure]. Probability (95% CI) of having an eGFR < 60 mls/min/1.73 m^2 ^at 2 years was 58.5% (35.1 - 76.0%) in the crescentic/mixed group. The sclerotic group all progressed to ESKD. Linear regression analysis demonstrated an association with slope of eGFR with baseline eGFR (p = 0.01), baseline proteinuria (p = 0.037) and need for dialysis (p < 0.001) after adjustment.

## Conclusion

We demonstrate the clinical utility of the new new histopathologic classification system and its ability to clearly discriminate outcomes among paediatric ANCA GN patients. Additional factors predicting outcome include baseline eGFR and dialysis. The new classification can be adopted for both clinical use and research studies.

## Disclosure of interest

M. Twilt: None Declared, D. Noone: None Declared, W. Hayes: None Declared, P. Thorner: None Declared, S. Benseler: None Declared, R. Parekh: None Declared, R. laxer Grant/Research Support from: Novartis, D. Hebert: None Declared.

